# Pressure-induced structural change in liquid GaIn eutectic alloy

**DOI:** 10.1038/s41598-017-01233-1

**Published:** 2017-04-25

**Authors:** Q. Yu, A. S. Ahmad, K. Ståhl, X. D. Wang, Y. Su, K. Glazyrin, H. P. Liermann, H. Franz, Q. P. Cao, D. X. Zhang, J. Z. Jiang

**Affiliations:** 1 International Center for New-Structured Materials and Laboratory of New-Structured Materials, State Key Laboratory of Silicon Materials & School of Materials Science and Engineering, Zhejiang University, Hangzhou, 310027 P. R. China; 2 Department of Chemistry, Building 207, Technical University of Denmark, DK-2800 Lyngby, Denmark; 3 Photon Science, Deutsches Elektronen-Synchrotron (DESY), D-22603 Hamburg, Germany; 4 State Key Laboratory of Modern Optical Instrumentation, Zhejiang University, Hangzhou, 310027 P. R. China

## Abstract

Synchrotron x-ray diffraction reveals a pressure induced crystallization at about 3.4 GPa and a polymorphic transition near 10.3 GPa when compressed a liquid GaIn eutectic alloy up to ~13 GPa at room temperature in a diamond anvil cell. Upon decompression, the high pressure crystalline phase remains almost unchanged until it transforms to the liquid state at around 2.3 GPa. The *ab initio* molecular dynamics calculations can reproduce the low pressure crystallization and give some hints on the understanding of the transition between the liquid and the crystalline phase on the atomic level. The calculated pair correlation function *g*(*r*) shows a non-uniform contraction reflected by the different compressibility between the short (1st shell) and the intermediate (2nd to 4th shells). It is concluded that the pressure-induced liquid-crystalline phase transformation likely arises from the changes in local atomic packing of the nearest neighbors as well as electronic structures at the transition pressure.

## Introduction

Phase transition in materials can be induced by varying external parameters, such as the temperature, pressure, electric or magnetic fields. Pressure-induced phase transitions in crystalline materials normally take place by dramatically structural changes, which can be unambiguously detected by diffraction experiments. In contrast, only local atomic structural characteristics with different short-range order (SRO) such as the coordination numbers and interatomic distances can be depicted if phase transition occurs in liquids and amorphous materials due to the absence of long-range order. Hence, the study of phase transition in liquids is still a great challenge. Although numerous experimental and theoretical researches in understanding of high-pressure effects on metallic liquids have been so far reported^1–13^, the atomic structures and the mechanism of pressure induced phase transitions, either crystallization or liquid-to-liquid phase transitions (LLPT), still remain poorly understood. Also, the experimental data have to be restricted to low pressures due to difficulties in obtaining metallic melts under extreme conditions, although recently developed laser-heated diamond anvil cells and laser shock compression have undergone rapid development for reaching ultrahigh P-T conditions^13–15^.

Metallic glasses (MGs) or amorphous alloys obtained by rapid quenching from melts can be regarded as ‘frozen liquid’. A large number of experiments and calculations have been performed in search of pressure-induced phase transition and transition mechanism in MGs. For example, amorphous-to-amorphous transitions (AATs) induced by pressure was surprisingly observed in Lanthanide-based MGs^16–20^, in which the structural transition of low-to-high density polyamorphism was suggested to be driven by *f* electron delocalization under high pressure. Another AAT was detected in main-group non-f-electron-containing Ca-Al MG. The origin of this polyamorphism was ascribed to the charge transfer from s and p-orbitals to d-orbitals of Ca under pressure^21^. Wu *et al*.^22^ re-explained the AAT in Ca-Al and pointed out that the enhancement of covalent interactions between Ca 3d and Al 3p electrons plays a key role in the occurrence of this AAT. Phosphorus^23^ and carbon^24, 25^ are good examples of high-pressure LLPT. But for metallic melts, can the LLPT take place under pressure? So far, only few reports concerning the pressure dependence of anomalous structural change and thermodynamic behaviors of metallic liquids have occurred. For example, the pressure induced LLPT was detected in monatomic liquid metal Ce by X-ray diffraction (XRD)^10^. More recently, Lee^12^ predicated a possible LLPT in the liquid Ti at high temperature by computer simulations. As a promising LLPT candidate, Gallium with low melting point attracts wide attention due to its peculiar physical properties and polymorphism in crystals^26–29^. The sudden change in electrical conductivity corresponds to the phase transition under compression^1^. The Ga_85.8_In_14.2_ eutectic alloy (hereafter marked as GaIn) with low melting point (288.3 K) has triggered considerable interest^30–34^, making it possible to probe the phase transition under high pressure. In this work, we present that the GaIn alloy exhibits two transformations under compression: one is a pressure-induced liquid-to-crystalline transition at 3.4 GPa, and the other is a crystal-to-crystal polymorphic transition from monoclinic to more complex triclinic phase near 10.3 GPa. To determine whether the liquid under low pressure experiences a liquid-to-liquid transition prior to crystallization, *ab initio* molecular dynamics (AIMD) calculations are performed to reveal the atomic and electronic structure changes in GaIn liquid alloy under pressure.

## Results

**Figure 1:**
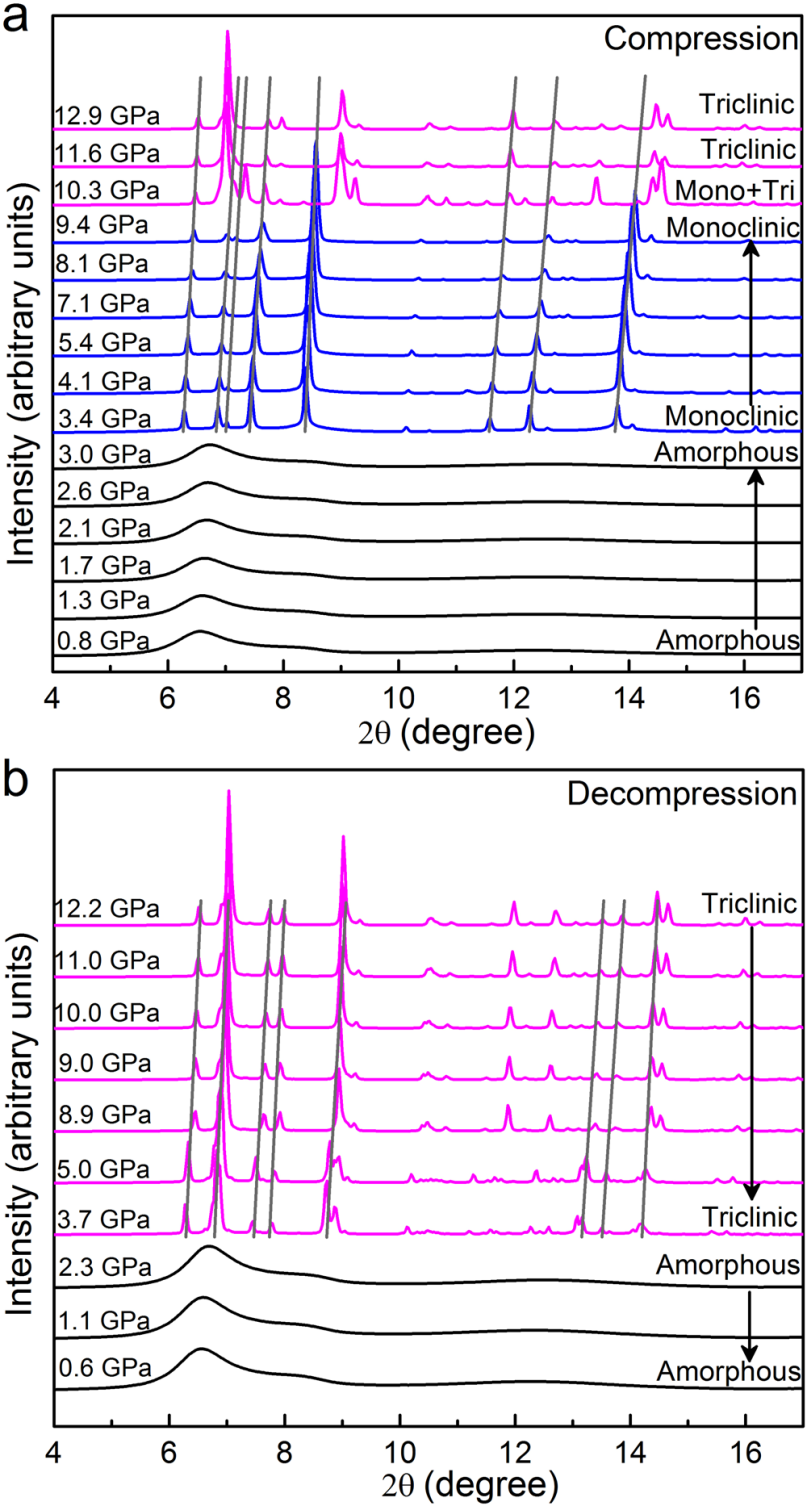
*In-situ* XRD patterns of Ga_85.8_In_14.2_ eutectic alloy upon (**a**) compression and (**b**) decompression in the pressure range of 0–13 GPa at ambient temperature. The light lines trace the change of Bragg peaks with pressure.

*In-situ* high-pressure XRD patterns for the liquid GaIn eutectic alloy up to ~13 GPa at room temperature was performed. Figure 1 shows all diffraction patterns of the GaIn alloy collected at room temperature over the entire compression and decompression processes for pressures ranging from ambient up to ~13 GPa then back down. As shown in Fig. 1a, the liquid phase is relatively stable upon compression to 3.0 GPa without obvious changes. By increasing the pressure to 3.4 GPa, sharp Bragg peaks are observed, corresponding to the low-pressure crystalline phase with C-centered monoclinic structure (a = 5.833(1), b = 8.797(1), c = 3.475(1) Å, β = 103.83(1)°) as indexed by using an program TREOR^35^. Further increase in pressure to 10.3 GPa results in the appearance of some extra Bragg peaks located at about 7.02, 9.02, 13.44 and 14.44 degree, indicating polymorphic transition. The high pressure crystalline phase displays a triclinic structure with an unit cell parameter of a = 4.618(1), b = 9.459(1), c = 2.547(1) Å, α = 90.30(1), β = 91.53(1), γ = 89.20(1)°. Upon further compression, the high-pressure crystalline phase remains stable up to the highest pressure of 12.9 GPa in this work. During subsequent decompression as shown in Fig. 1b, the high-pressure crystalline phase remains unchanged until it transforms back to the amorphous-like and/or liquid-like phase at ~2.3 GPa. To confirm the final phase transition during decompression is just a melting process, we carefully examine all the structure factors *S*(*q*) of the GaIn alloy before crystallization during compression and after vitrification during decompression from experimental and theoretical points of view.

## Discussion

**Figure 2:**
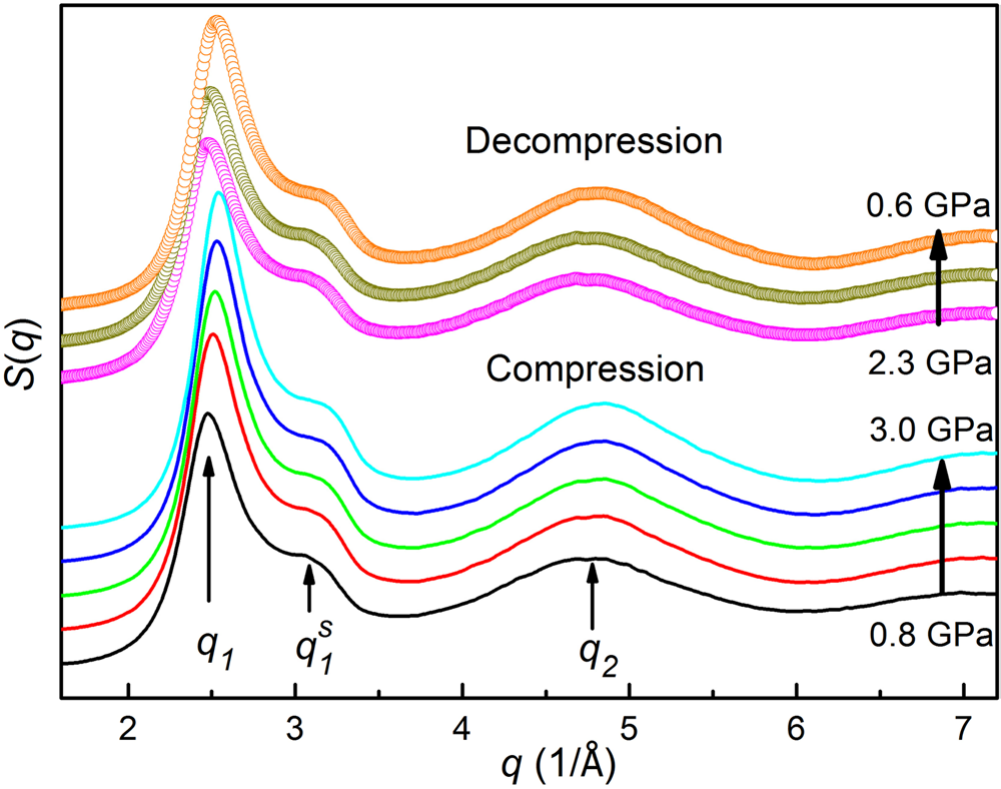
Structure factors *S*(*q*) of Ga_85.8_In_14.2_ alloy obtained by XRD before crystallization during compression (lower part, solid lines by a shift in y axis) and after vitrification during decompression (upper part, circles by a shift in y axis). The arrows show the principle peaks (*q*_*1*_ and *q*_*2*_) as well as the small sub-peak (*q*_*1*_^*s*^) separated from the first main peak.

**Figure 3:**
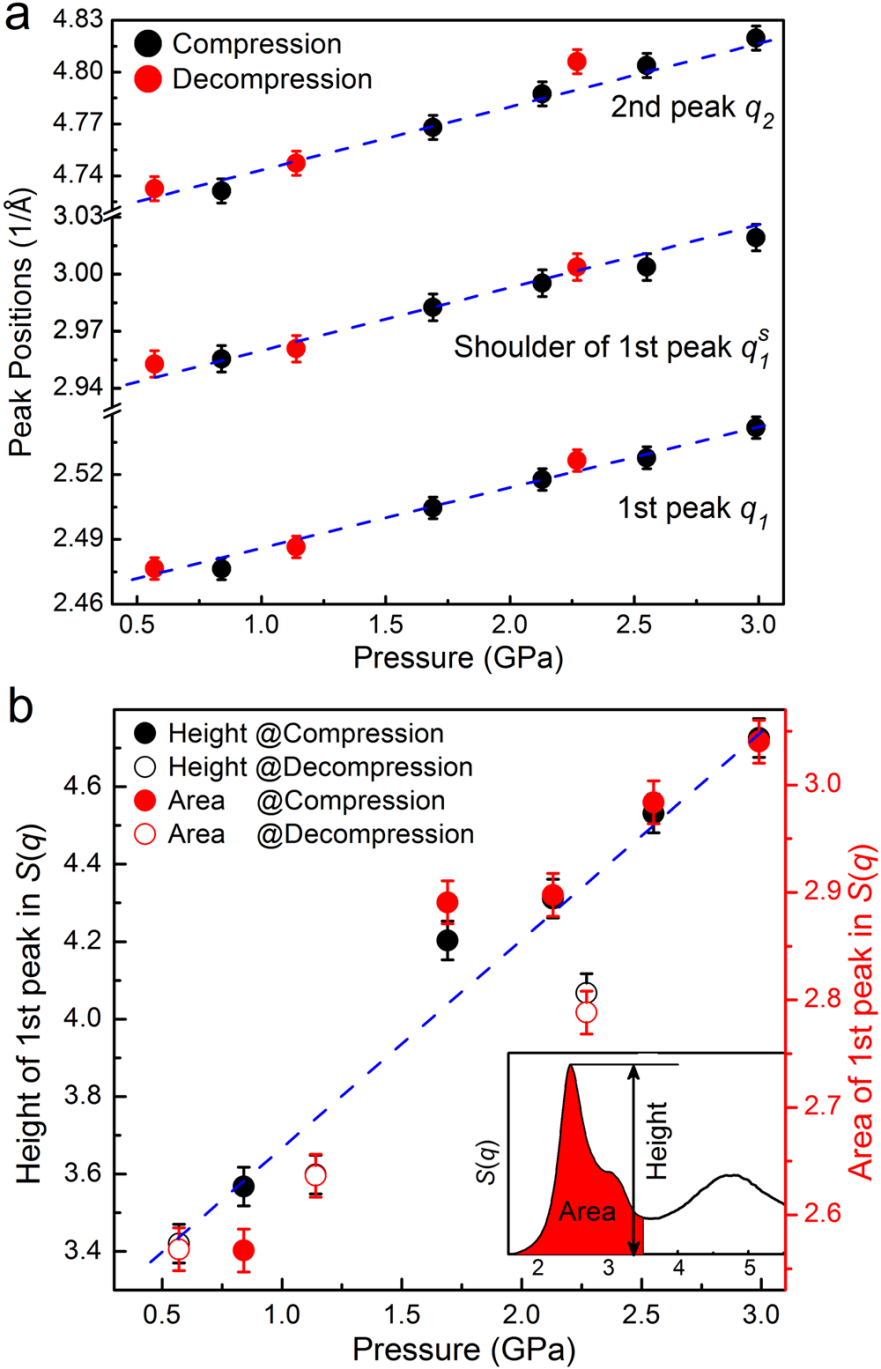
The 1^st^ and 2^nd^ peak positions in *S*(*q*) as a function of pressure prior to crystallization upon compression and after vitrification during decompression, in which the first peak is fitted by two Gaussian functions. (**b**) Pressure-induced changes of height and area (or intensity) in the first peak of *S*(*q*), as indicated in the inset.

Evidently, each *S*(*q*) as shown in Fig. 2 consists of a maximum located at *q* ≈ 2.5 Å^–1^ with a shoulder on the right side at *q* ≈ 3.2 Å^–1^ and a second hump at *q* ≈ 4.8 Å^–1^. With increasing pressure, *S*(*q*) shifts towards high *q* without a change in the shape of the profile, as expected for pressure-induced densification. Simultaneously, the first peak positions (main component *q*_*1*_ and shoulder *q*_*1*_^*s*^) as well as the second peak position (*q*_*2*_) all increase with pressure at almost the same rate as shown in Fig. 3a. The pressure-dependent height and area (or intensity) of the first peak in *S*(*q*) are plotted in Fig. 3b, following similar trends prior to crystallization upon compression and after vitrification during decompression, i.e., the higher the pressure, the higher the height and intensity. These results confirm that the disordered structures of the GaIn alloy obtained upon compression below ~3.4 GPa and decompression below ~3.7 GPa are of the similar liquid structure. No liquid-to-liquid phase transition occurs for the eutectic GaIn liquid alloy in the studied pressure range at ambient temperature.

**Figure 4:**
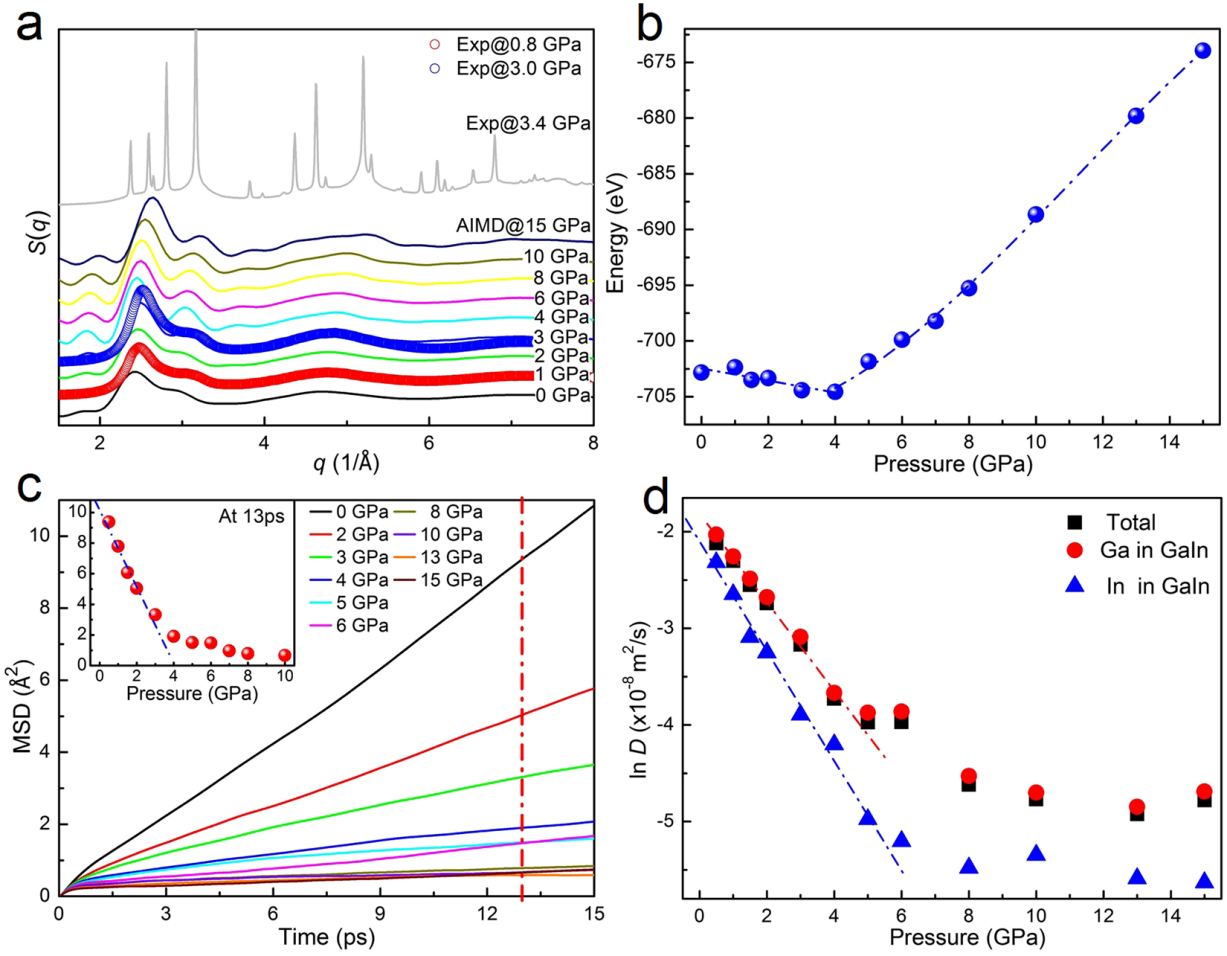
Structural information and dynamic behaviors of Ga_86_In_14_ alloy obtained from AIMD method. (**a**) Comparison of the calculated and experimental total structure factor *S*(*q*) at selected low-pressures. (**b**) The pressure-energy relation of GaIn alloy. (**c**) Time dependence of the mean square displacement (MSD) with pressure, the left inset showing the MSD located at 13 ps. (**d**) Self-diffusion coefficients, *D*, for total, Ga and In atoms, respectively.

The local structure and the pressure-dependent structural evolution during liquid-to-crystalline phase transition were further studied by *ab initio* molecular dynamics calculations. However, it should be noted that of the finite-size effect and limited simulation time of the theoretical calculations, AIMD results can not completely reproduce the experimental results. Nevertheless, these results clearly reveal the solidification process in the liquid GaIn alloy from an atomic-level scale point of view. Figure 4a shows the calculated structure factors *S*(*q*) at selected pressures, which are consistent with those from experiments in the low pressure range, providing reasonable configurations for further structural analyses. The system energy versus pressure in Fig. 4b can be clearly divided into two regions: below ~4 GPa, it slightly decreases during compression, and the other above ~4 GPa it increases with pressure. An abrupt kink emerged at a pressure of ~4 GPa. The calculated mean-square displacements (MSD) and diffusion coefficients at different pressures are given in Fig. 4c,d, respectively. Below ~4 GPa MSD is linear with time *t*, confirming that the system is in the liquid state. By increasing pressure, MSD becomes very small, in particular at 13 ps as shown in the inset of Fig. 4c, where an obvious kink emerges near ~4 GPa. The pressure dependence of the diffusion coefficients of total and individual components (Ga and In atoms) clearly reveal that the slopes below and above ~5 GPa are significantly different. Such differences in system energy, MSD and diffusion coefficients all demonstrate that a phase transition occurs at 4~5 GPa. In addition, the atomic movement of In atoms is slower than that of Ga atoms at the same pressure.

**Figure 5:**
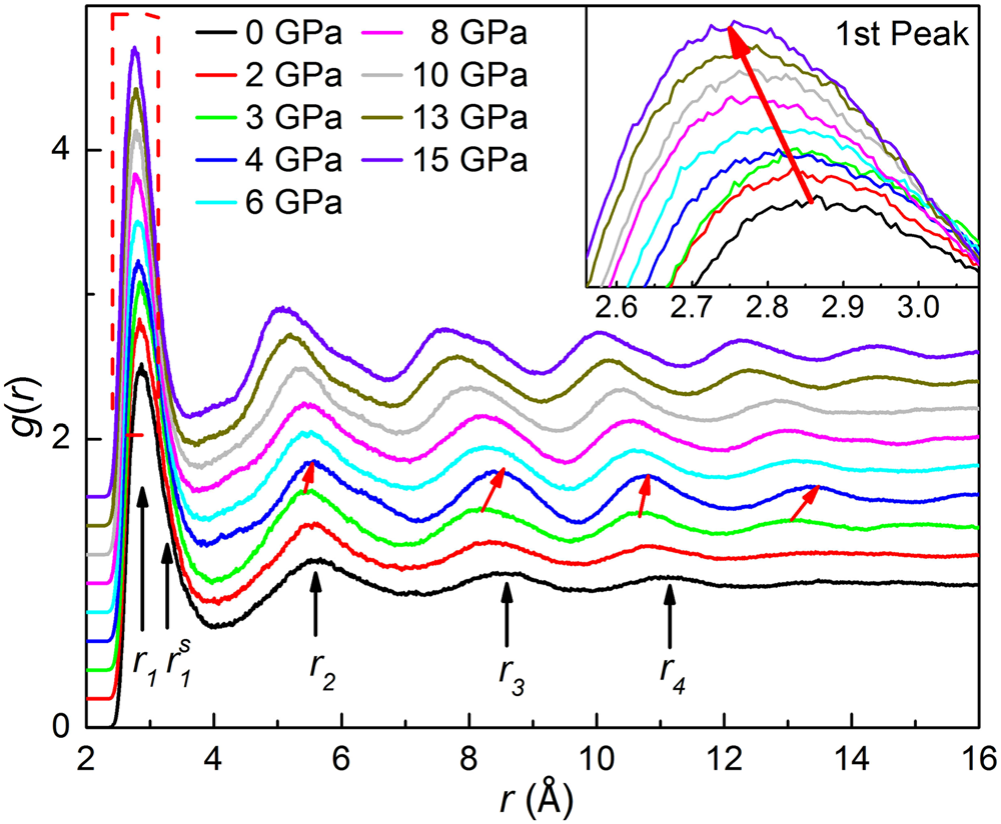
The pressure dependence of pair correlation function *g*(*r*) as calculated with AIMD method. The inset shows the magnification of the first peak in *g*(*r*). The red arrows denote sudden jumps in peak positions of the 2^nd^ to 4^th^ shells between 3 and 4 GPa.

**Figure 6:**
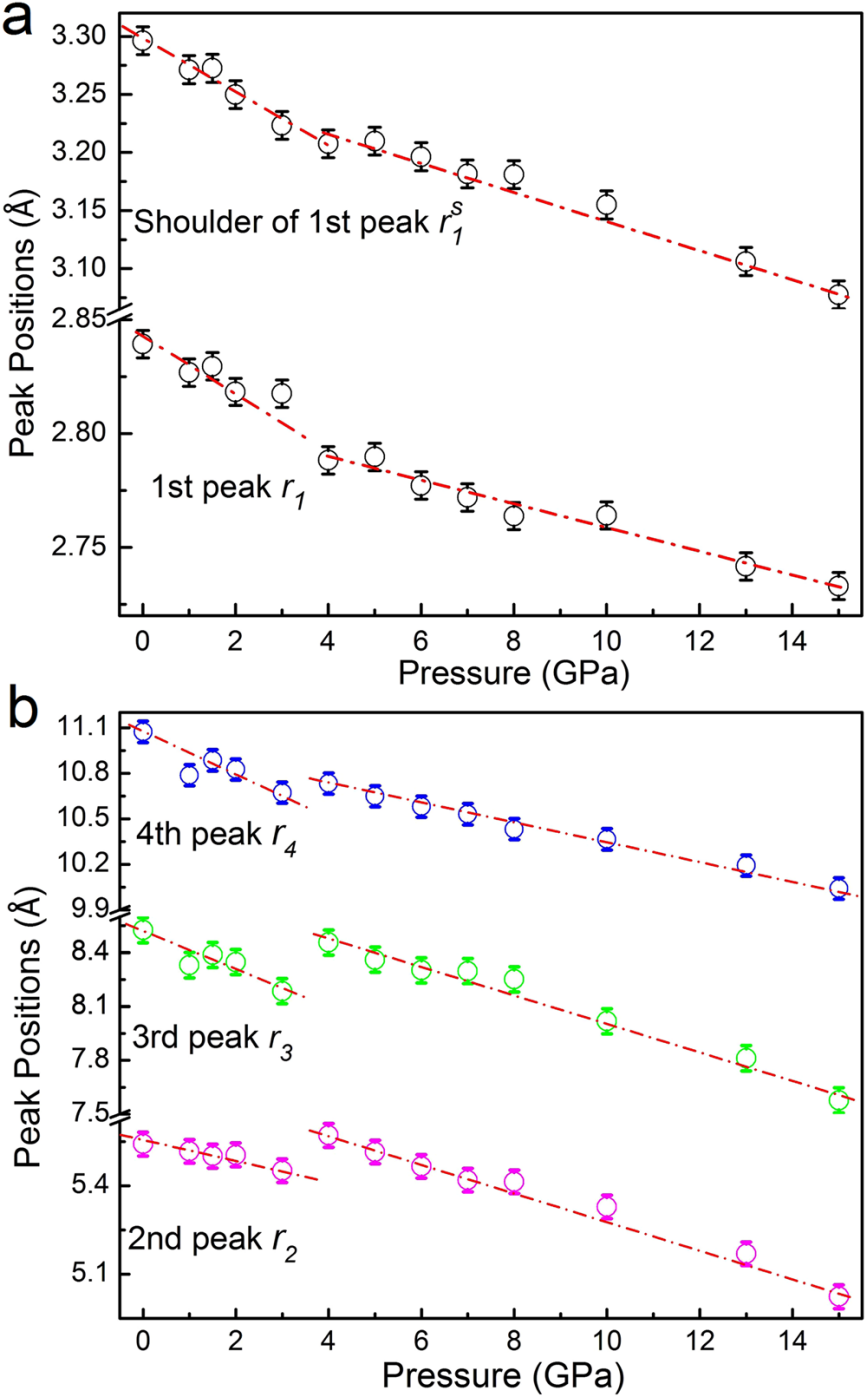
The first four peak positions of *g*(*r*) obtained from AIMD upon compression for the Ga_86_In_14_ eutectic alloy.

To explore more structural information associated with the transition, the pressure dependence of the pair correlation functions *g*(*r*) obtained from AIMD is illustrated in Fig. 5. Upon compression to ~3 GPa, all peaks in *g*(*r*) move to lower *r* values and are slightly sharpened, indicating the densification of liquid GaIn. The far nearest (i.e., fourth and fifth) neighbor peaks become more pronounced, implying the promotion of short and intermediate-range order in the liquid. With further increase in pressure to ~4 GPa, surprisingly, a kink and jump are detected in the first peak position and far nearest neighbor peak positions in Fig. 6a,b, respectively, indicating a sudden structural change and an anomalous expansion during solidification, analogous to the behavior of pure Ga, i.e., the density decreases upon solidification^36, 37^. Furthermore, two small peaks located at ~4.3 and ~6.5 Å start to emerge, reflecting the onset of crystallization. We carefully examine the densification process of liquid GaIn below ~4 GPa by calculating a reduced position of the first main peak in *S*(*q*) in Fig. 3a and reduced positions of the first four peaks in *g*(*r*) in Fig. 6, listed in Table 1. It is known that the slope of *q*_1_(*P* = 0)/*q*_1_(*P*) reflects the linear compression of the whole liquid during compression, which is much larger than those of *r*_1_(*P*)/*r*_1_(*P* = 0) and *r*_2_(*P*)/*r*_2_(*P* = 0), and close to the slopes of *r*_3_(*P*)/*r*_3_(*P* = 0) and *r*_*4*_(*P*)/*r*_*4*_(*P* = 0). These results reveal the lower compressibility for atoms on the first and second shells and higher compressibility for the third and fourth shells in liquid GaIn alloy during compression.

**Table 1:** Ratio parameters from experimental *S*(*q*) and calculated *g*(*r*) of liquid GaIn alloy changing with pressure *P* during compression below 4 GPa.

Ratio vs. *P*	*q*_1_(*P* = 0)/*q*_1_(*P*)	*r*_1_(*P*)/*r*_1_(*P* = 0)	*r*_2_(*P*)/*r*_2_(*P* = 0)	*r*_3_(*P*)/*r*_3_(*P* = 0)	*r*_4_(*P*)/*r*_4_(*P* = 0)
Slope	−0.011	−0.004	−0.005	−0.012	−0.011

*q*_1_: first-peak position of experimental *S*(*q*); *r*_1_, *r*_2_, *r*_3_ and *r*_4_: the first four peak positions of calculated *g*(*r*), respectively.

**Figure 7:**
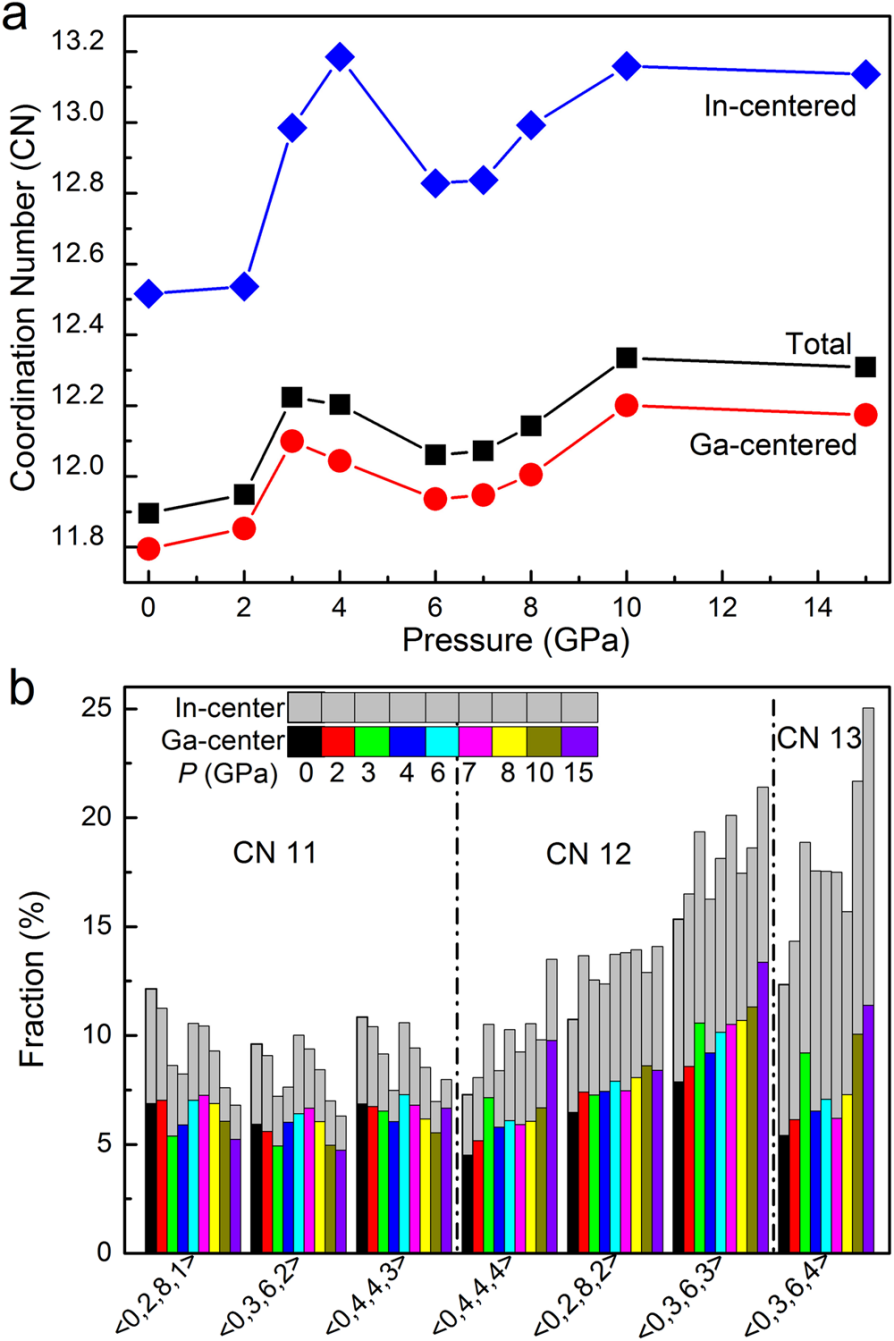
The structural features obtained from Voronoi tessellation (**a**) The calculated nearest-neighboring total and partial coordination numbers (CNs) with pressure in Ga_86_In_14_ alloy. (**b**) The distribution of the most abundant Voronoi polyhedra around Ga and In atoms in Ga_86_In_14_ alloy during compression.

We further used Voronoi tessellation method^38, 39^ to characterize the local atomic packing and determine the nearest-neighboring coordination number (CN), in which the three-dimensional space is divided into polyhedral cells constructed by a center atom and its nearest-neighboring atoms. This cell can be expressed by a set of indices <n_3_, n_4_, n_5_, n_6_>, specifically, n_i_ is the number of *i*-edged polygon. The total number of faces on the Voronoi polyhedron is equivalent to the coordination number (CN) for a selected central atom. Figure 7a shows the calculated average total, Ga-centered and In-centered coordination numbers (CNs) in the GaIn alloy. Upon compression, the three average CNs show similar trend, i.e., they first increase with pressure, then at ~4 GPa, suddenly drops, afterward, they further slightly increase with pressure. These sudden drops at ~4 GPa are attributed to the liquid-to-crystalline phase transition by abrupt change in atomic arrangement. The average CN for the In-centered polyhedra is found to be larger than that for Ga-centered ones, which might be linked with relatively large atomic radius of In atoms. The distribution of the most abundant Voronoi polyhedra (VP) around Ga and In atoms are depicted in Fig. 7b at critical pressures. The dominant VPs around In atoms are CN = 12 (<0, 3, 6, 3>, <0, 2, 8, 2>) and CN = 13 (<0, 3, 6, 4>), while around Ga atoms, many VPs (CN = 11, 12, and 13) have similar fractions. During compression, relative fractions for CN = 11 VPs decrease while those for CN = 12 and 13 VPs increase below ~4 GPa, esp. for In-centered VPs. Above ~4 GPa, such trend becomes unclear. It is known that <0, 3, 6, 3> and <0, 3, 6, 4> VPs are assigned to be distorted-FCC polyhedra, which have high fractions in both liquid state below ~4 GPa and crystalline state above ~4 GPa.

**Figure 8:**
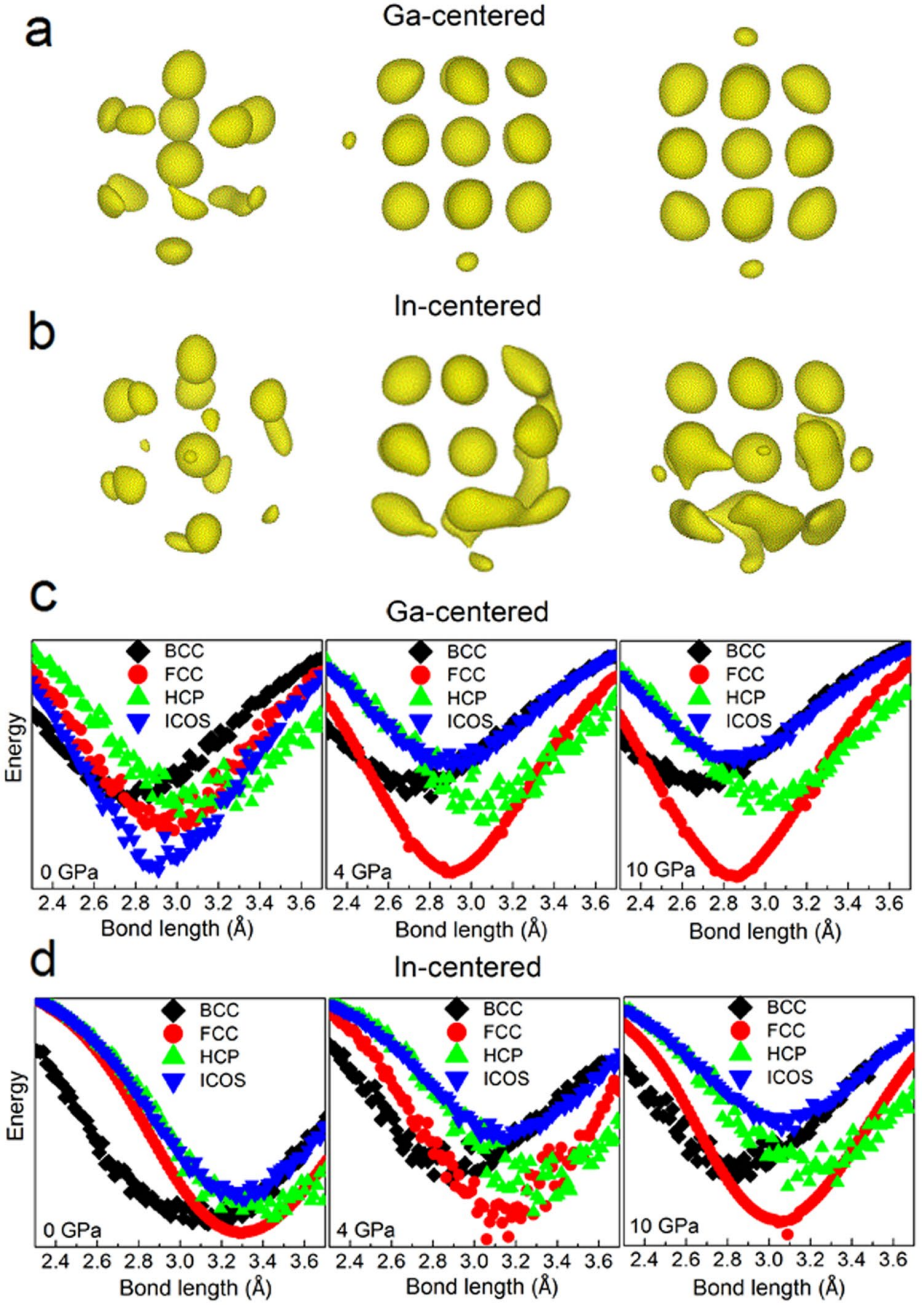
The cluster alignment results of Ga_86_In_14_ alloy at selected pressures. (**a**,**b**) are atomic-density contour plots of final configurations of collective alignment separately for Ga and In-centered local structures, respectively. (**c**,**d**) are attractive potential energies as the function of bond-length curves for BCC, FCC, HCP, and icosahedral (ICOS) SROs in Ga and In centered clusters, respectively.

In order to further explore the nature of the structural change associated with the pressure-induced transitions in GaIn alloy, an atomistic cluster alignment (ACA) method^40^ was used, in which local environment of each atom is characterized by an atomic cluster including the atom itself and its nearest-neighbors up to a distance of the first minimum in *g*(*r*). One cluster containing 16 atoms extracted from 2000 configurations is aligned by the rigid rotation and translation of the clusters with respect to each other, eventually giving visual information about the local structure features. The details of the ACA method can be found in ref. 40. Since different types of central atoms may have different local structural orders, and therefore the collective alignments are separately performed for local clusters centered by Ga and In atoms. Figure 8a,b illustrate the atomic-density contour plots at three representative pressures, providing visually resolved atomic distribution around Ga and In atoms, respectively. The topological ordering in the liquid state at 0 GPa is different from the crystalline state at 10 GPa. The local environment of Ga is relatively more ordered than that of In. For example, at 0 GPa, a discernible icosahedron-like pattern for the Ga-centered cluster in Fig. 8a is identified. At 4 GPa and 10 GPa, the Ga-centered and In-centered aligned clusters show highly FCC-like symmetry, suggesting the formation of long-range order. Sequentially, the alignment between collective-aligned clusters and typical BCC, FCC, HCP and ICOS templates have also been performed to yield the potential energy versus bond-length curves. The template-alignment results for Ga and In at three pressures are shown in Fig. 8c,d, respectively. Figure 8c shows that at ~0 GPa the energy curve for icosahedral short range order in Ga centered clusters is slightly lower than other structures, implying a relatively stronger icosahedron-like SRO in GaIn liquid at room temperature, while FCC-like order becomes stable with increasing pressure to 4 GPa and even 10 GPa corresponding to the crystallization process. The same conclusion can be drawn from the results of In clusters in Fig. 8d. The FCC-like SRO starts to emerge from disordered liquid at 4 GPa and grows slowly with the increase of pressure. Therefore, we speculate that although the local atomic structure gradually displays FCC-like symmetry with pressure, the interplay between Ga and In atoms and different degrees of distortion caused by atomic sizes may favor the final formation of monoclinic phase observed in the experiment in Fig. 1.

These results obtained from AIMD calculations confirm that below ~4 GPa, liquid GaIn is densified with pressure, i.e., average CNs for total, Ga-centered and In-centered VPs increase and local atomic bonds (first-fourth nearest neighbors) decrease. At ~4 GPa, a liquid-to-crystalline phase transition occurs, reflected by a kink in the slope of pressure dependent position of the first peak in *g*(*r*), sudden jumps in positions of the second, third and fourth peaks in *g*(*r*), and drops in CN for total, Ga-centered and In-centered VPs, as observed in experiments in Fig. 1.

**Figure 9:**
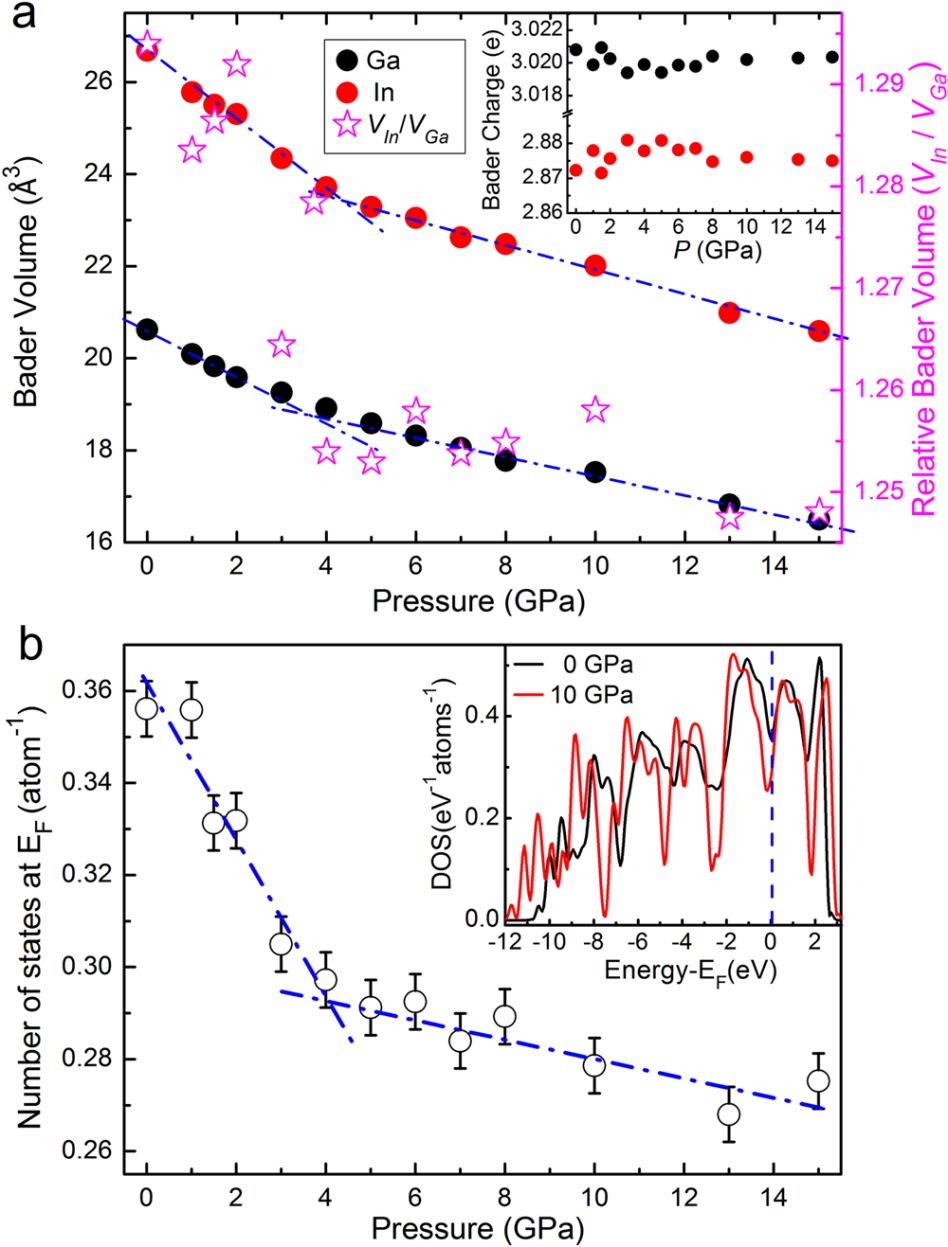
Theoretical Bader analysis and electronic density of states (DOS) for Ga_86_In_14_ alloy. (**a**) The pressure dependence of Bader volume, the relative Bader volume of In to Ga, and Bader charge (inset). The blue dash dots are drawn to guide the eyes. (**b**) The DOS at the Fermi level as a function of pressure. The inset shows the total DOS of GaIn alloy at ambient pressure and 10 GPa. The Fermi level is set to 0.0 eV.

Gallium has been extensively studied for anomalous melting with a density increase of 3.2% and a big difference in the electronic structure between the liquid phase (l-Ga) and the solid (α-Ga crystal)^1, 41^. The l-Ga shows nearly free-electron-like behavior, while the electronic density of states (DOS) of α-Ga has a pronounced pseudo-gap at the Fermi energy (E_F_) attributed by the coexistence of metallic and partial covalent bonds. However, for the same group element In, no such anomalous density change during melting was reported, and the electric structure are rather similar between the liquid and crystalline phases^1^. Recently, Wu^22, 42^ and Lou^21^ have demonstrated that the electronic structure plays a key role in polymorphic transitions in Ce-Al and Ca-Al MGs. To shed light on electronic effect on the solidification of GaIn eutectic alloy, density functional theory calculations (DFT) were performed to characterize electronic structure of the liquid and crystalline GaIn alloy. Here, a simple approach of partitioning the charge via Bader analysis^43, 44^ was performed on a 180 × 180 × 180 grid after obtaining the equilibrated configurations. In this method, space was divided into atomic regions as Bader volume based purely on the charge density. The dividing surfaces (also called zero-flux surfaces) separating these volumes are at a minimum in the charge density, and the charge enclosed within the Bader volume can be integrated radially from the charge density maximum to the surface. Specifically, Bader volumes was used to define atomic size. Both the Bader analysis and total DOS obtained by averaging twenty configurations are presented in Fig. 9. As shown in Fig. 9a, clear kinks in the pressure dependent Bader volume for both Ga and In are detected at the pressure ~4 GPa. The slopes of the pressure dependence of Bader volume of Ga and In above ~4 GPa are lower than those below ~4 GPa. Besides, the *V*_*In*_/*V*_*Ga*_ Bader volume ratio suddenly declines upon compression at around 4 GPa in Fig. 9a. The Bader charge in the inset of Fig. 9a shows that at ambient pressure an average electron charge of ~0.13e transfer from In to Ga is found, but varying slightly upon compression. This shows that the Bader charge is not very sensitive to topological order but non-linear compression behavior of Bader volume adjusts atomic size ratio. As a result, atomic packing facilitates the redistribution of the coordination polyhedral and favors the crystallization. To further acquire the information of electronic structure in the liquid and the crystalline states, the calculated total electronic density of states at two representative pressures of 0 and 10 GPa are shown in the inset of Fig. 9b. We can see that the peaks in DOS become more sharpened upon compression which originate from the more overlaps of electronic states among atoms. DOS of liquid GaIn alloy at 0 GPa has a shallow gap at the Fermi level, indicating a strong metallic-like bonding, while a rather deep minimum at E_F_ is detected at 10 GPa as expected for pure α-Ga crystal. The DOS at E_F_ as a function of the pressure is also plotted in Fig. 9b, which clearly reveals a kink at the pressure of the liquid-to-crystalline phase transition of ~4 GPa, reflecting the obvious change not only in short-range order but also electronic structures during crystallization. Hence, it is reasonable to assume that high-pressure induced crystallization in the liquid GaIn alloy is affected not only by the changes in the atomic arrangements but also in the electronic structures.

In summary, we report the results of a diamond anvil cell (DAC) synchrotron x-ray study on liquid Ga_85.8_In_14.2_ eutectic alloy and experimentally elucidate that the liquid undergoes pressure-induced crystallization at about 3.4 GPa and subsequent polymorphic transition from monoclinic to triclinic modifications near ~10.3 GPa. Upon decompression, the high-pressure crystallization phase remains stable until it transforms back into the final liquid state at ~2.3 GPa. *Ab initio* molecular dynamics calculations can reproduce the low-pressure crystallization process and elucidates the structure, dynamic, and electronic properties for the liquid-to-crystalline phase transition. It is found that upon compression, the liquid is compressed in a rate much faster than the contraction of the first and second nearest neighbor shells, but similar to those of the third and fourth shells. This non-uniform contraction at local atomic levels causes atomic rearrangement, consequently resulting in a liquid-to-crystalline phase transition at ~4 GPa. It reveals that this transition is accompanied with (1) kinks in the pressure-dependent position of the first four peaks in *g*(*r*), Bader volume of Ga and In atoms, and electronic density of states at the Fermi level; (2) jumps in the pressure-dependent positions of the second, third and fourth peaks in *g*(*r*); and (3) drops in coordination numbers of total, Ga-centered and In-centered clusters. This pressure-induced liquid-to-crystalline phase transformation likely arises from the changes in local atomic packing as well as changes in the electronic structure at the transition pressure.

## Methods

### Samples preparation and the *in-situ* high pressure X-ray diffraction experiment

The sample of GaIn eutectic alloy used here was prepared with high purity Ga (99.99%) and In (99.99%), and then was loaded in symmetric diamond anvil cell (DAC) for *in-situ* high pressure synchrotron XRD. The sample chamber was made by drilling a hole (~150 µm in diameter) in T301 steel gasket. The sample chamber was filled with the liquid along with a tiny piece of ruby as a pressure-calibrator. High pressure XRD at ambient temperature was performed at beamline P02.2 of PETRAIII with the energy of 42.7 keV. The DAC apparatus was used to generate pressure and the ruby fluorescence method was used for pressure calibration^45^. XRD data were collected and recorded by a Perkin Elmer XRD1621 detector. After integration of the 2D imaging plate, the raw 1D diffraction spectra were obtained and converted into structure factors using the program PDFgetX2^46^.

### *Ab initio* molecular dynamic simulation

The AIMD simulation of GaIn alloy in canonical NVT ensemble was performed based on the density functional theory (DFT) by using the Vienna Ab initio Simulation Package (VASP) together with projector augmented wave (PAW) potentials and generalized gradient approximation (GGA) exchange correlation functional^47–49^. Only Γ point was used to sample the Brillouin zone of the supercell and Nosé-Hoover thermostat for temperature control^50^. The Verlet algorithm was used to integrate Newton’s equations of motion and the time step of ion motion was 3 fs. A supercell containing 250 atoms (215 Ga atoms and 35 In atoms randomly distributed in a cubic box) with periodic boundary conditions was prepared and thermally equilibrated at 1500 K for more than 18 ps. The system was then quenched in steps to 300 K with a cooling rate of 0.1 K/step, followed by the isothermal relaxation for more than 30 ps. Based on the equilibrium liquid state, the thermodynamic external pressure was obtained by gradually adjusting system volume at 300 K. At each pressure point, additional 30 ps were adopted after pressure equilibration and the data of last 12 ps were collected for statistical analyses.
